# Stearic-Acid-Coated Sand: A Game Changer for Agriculture Water Management

**DOI:** 10.3390/nano15100721

**Published:** 2025-05-11

**Authors:** Muhammad Abdullah, Mergen Zhazitov, Nazerke Kydyrbay, Tolagay Duisebayev, Yerbolat Tezekbay, Olzat Toktarbaiuly

**Affiliations:** Renewable Energy Laboratory, National Laboratory Astana (NLA), Nazarbayev University, Kabanbay Batyr 53, Astana 010000, Kazakhstan; muhammad.abdullah@nu.edu.kz (M.A.); mergen.zhazitov@nu.edu.kz (M.Z.); nazerke.kydyrbay@nu.edu.kz (N.K.); tolagay.duisebayev@nu.edu.kz (T.D.); yerbolat.tezekbay@nu.edu.kz (Y.T.)

**Keywords:** superhydrophobic coating, SACS, surface modification, functionalized sand

## Abstract

This study presents the synthesis, characterization, and evaluation of stearic-acid-coated sand (SACS) as a superhydrophobic material for agricultural water management applications. The fabrication process involves coating silica sand particles with stearic acid in an ethanol-based solution, followed by controlled drying to achieve a stable and uniform hydrophobic layer. Structural, chemical, and physical characterizations confirmed the successful functionalization of the sand surface. The coated sand exhibited a high water contact angle (WCA > 150°), indicating strong water repellency and potential for reducing water loss in soil systems. Experimental results demonstrated enhanced moisture retention in SACS-treated soil, prolonging water availability by up to four additional days compared to untreated samples. Despite its promising performance, potential degradation under acidic or organic solvent exposure remains a concern for long-term application. Overall, this work presents SACS as a low-cost, scalable solution to improve water conservation in dry agricultural areas, supporting sustainable farming practices.

## 1. Introduction

Socio-ecological systems, agricultural productivity, and global supply chains are all widely impacted by worldwide water scarcity on a global basis. Water scarcity and climate change underpin vulnerabilities, especially in developing nations. Over 500 million individuals in vulnerable basins experience increased socio-ecological threats as a result of climate sensitivity [1]. Water scarcity in the Gulf Co-operation Council (GCC) region adversely affects agricultural production, food security, and economic stability, highlighting the importance of sustainable practices [2,3].

**Figure 1:**
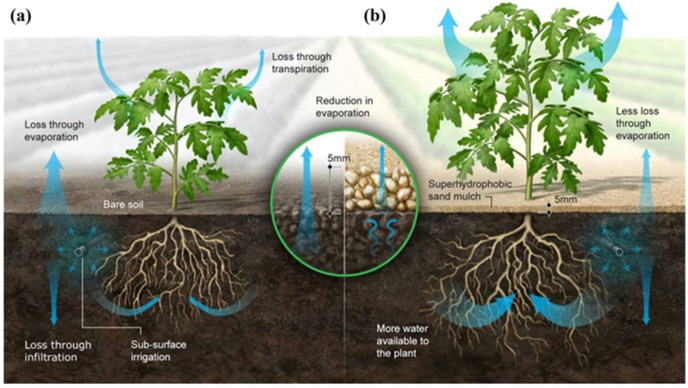
Comparison of water dynamics in unmulched and superhydrophobic sand mulched soil. (**a**) illustrates traditional unmulched soil, where substantial water is lost through evaporation and deep percolation, leading to inefficient water use and reduced availability for plant roots. (**b**) depicts the application of superhydrophobic sand mulch (SHS), which creates a dry diffusion barrier that significantly reduces evaporation and conserves moisture near the root zone, enhancing water availability and efficiency in arid regions [10].

Limited water supplies impede agricultural production, exacerbating poverty and deepening suffering, and thereby impacting rural livelihoods in arid regions profoundly [4]. In addition to its regional influence, water shortage endangers world supply chains, particularly vulnerable ones, making strategic management a crucial component of economic stability [5]. Agriculture consumes 70% to 90% of the globe’s freshwater, highlighting the imperative for improved water management practices. With a water consumption footprint estimated between 5938 and 8508 km^3^/year, regions like Pakistan—which uses 96% of its water in agriculture—are challenged with problems of inefficiency and wastage, requiring intervention in such regions [6,7]. Water footprint (WF) philosophy zeroes in on blue water (surface water and groundwater) as the driving force for crop production. Agricultural practice optimization can minimize water use, but climate and land-use change are projected to increase the world’s blue water footprint by 22% by 2090 [6,7]. There are also behavioral considerations that are essential; understanding agricultural water-use behavior is the first step to making it more efficient [8]. Regenerative and nature-inspired practices enhance water productivity and encourage long-term sustainability even more [9]. As suggested by Figure 1, super-hydrophobic technology like SHS and our recently developed SACS can take center stage in transforming agricultural water management in semi-arid and arid regions. Through evaporative loss reduction and the conservation of soil moisture, such technologies not only facilitate sustainable agricultural production, but also make agricultural operations financially sustainable in water-short situations [10,11].

Sand-textured soil has low nutrient and water retention [12,13], with quick percolation of water resulting in wasteful applications of irrigation and higher cost [14]. Tillage conservation and cover crops are techniques to conserve water-use efficiency through improving soil state and moisture retention [15]. Technologies like organic matter amendments and biochar significantly increase soil water and yield, and biochar increases grain yield by 51.6%. In addition, incorporating materials like Pisha sandstone reduces water seepage, improving the availability of moisture. Soil mixtures—for instance, adding clay to sandy soils—further increase water holding [16]. The use of traditional gravity surface irrigation methods is highly inefficient, with a lot of water lost, impeding agricultural productivity. Notably, 40–50% water loss via evaporation and runoff is normal; surface irrigation has only 50–60% application efficiency compared to 90–95% when improved systems like sprinkler and drip irrigation are employed [17]. Such inefficacies also limit crop yields, with traditional methods of irrigation yielding much lower outputs; direct-seeded rice, for example, has 15.1–21.1% greater yield when improved systems are employed [18]. Economically, high water wastage increases operational costs, making conventional irrigation unsustainable for farmers [19].

SACS is a new method to enhance water efficiency and retention in field conditions. By depositing a stearic acid coating on sand grains, SACS acquires hydrophobic characteristics, which improve moisture management in soil [20]. This method maximizes water retention capacities, minimizes rates of evaporation, and promotes plant growth by maintaining the availability of water to plant roots for significant lengths of time. The hydrophobic surface, due to the stearic acid coating, reduces water loss by evaporation, a critical advantage in moisture management [20]. SACS also enhances water retention and slow release in sandy soils via wettability and infiltration inhibition, with maximum hydrophobicity at approximately 5 g/kg amendment loading; its long-term field durability and relative efficacy compared to other amendments such as biochar are yet to be tested [21,22,23]. In addition, the altered physical properties of the sand improve the structure of the soil and therefore enable better aeration and root penetration [24]. New technologies, such as smart irrigation systems and water-saving technology, have been developed to optimize water use in agriculture for the purposes of productivity and sustainability [25,26]. Research has shown that water delivery and agri-supply chain integration can enhance efficiency and responsiveness in water management practices [27]. Studies indicate that the adoption of water-saving technology is influenced by several factors, including farm type, farmer education, and support from institutions [28]. The effect of agricultural water management (AWM) innovations will be quite different across geographical and climatic conditions, requiring focused approaches [28].

SACS significantly enhances the water retention capacity of soil. For instance, fatty acid still pitching can convert up to 98% of clay into water-stable aggregates, which improve water retention [29]. The hydrophobic and hydrophilic personalities that come together in SACS optimize water uptake and retention through the selective control of infiltration, evaporation, and slow release in a manner that is beneficial for the effective management of water in dry conditions and sandy soils with low indigenous retaining capacity [30]. Scalability, sustainability in the environment, and appropriateness for particular types of soils, however, are some of the areas where further growth and research are needed. Parallel investigations involving superhydrophobic coatings with reduced graphene oxide sources applied to other industries also present encouraging mechanisms to surpass such constraints [31,32]. The hydrophobicity of SACS decreases evaporation loss and makes more water accessible to crops [33]. Minimizing the loss of water is a great concern for agriculture in a sustainable context, especially in areas that are prone to drought. Studies have demonstrated that the use of coated fertilizers, such as those containing stearic acid, can lead to enhanced plant biomass and nutrient content and, as such, improve the overall productivity of crops [34]. There are limited studies on the application of SACS in agriculture, particularly its impact on soil productivity and crop yield.

Existing solutions often fail to integrate economic and ecological considerations, highlighting a need for more comprehensive approaches [35]. The overall objective of this work is to achieve a cost-effective, scalable, and stable pathway for the production of SACS to enhance water saving in dry agriculture conditions. We first synthesized SACS by surface-coating silica sand particles with stearic acid in an ethanol-based solution in order to create an even hydrophobic coating through controlled drying treatments. Second, we carried out comprehensive structural, chemical, and physical analyses of SACS via scanning electron microscopy (SEM), X-ray diffraction (XRD), Raman spectroscopy, Fourier-transform infrared spectroscopy (FTIR), and energy-dispersive spectroscopy (EDS) to verify the successful functionalization of the sand surface. Finally, we ascertained the water repellency of SACS via WCA measurements and tested its influence on soil moisture retention against untreated sand. Through these concerted efforts, our study demonstrates that SACS is capable of significantly reducing evaporative water loss and prolonging soil moisture availability, thereby offering a sustainable means of managing water scarcity in agricultural environments.

## 2. Materials and Methods

### 2.1. Chemicals

The chemicals used in SACS preparation were selected to achieve optimum hydrophobicity and long-term stability in agricultural water management. Stearic acid (95% pure C_17_H_35_COOH) was used as the primary surface modifier. Stearic acid was dissolved in ethanol (95% pure C_2_H_6_O) to achieve satisfactory uniformity of the coating slurry, since ethanol possesses satisfactory solubility for stearic acid and supports the coating process. Distilled water was added whenever needed to create a low-energy solution. Silica sand, which was chosen based on its stability and ready availability, was employed as the substrate.

### 2.2. Preparation of Stearic-Acid-Coated Sand (SACS)

**Figure 2:**
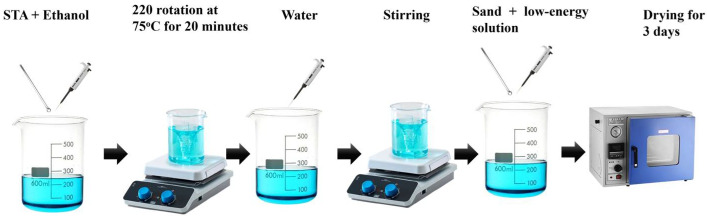
Fabrication of stearic-acid-coated sand (SACS).

The SACS manufacturing process is demonstrated in Figure 2. Initially, the stearic acid ethanol solution was made at 75 °C. For the purpose of this work, 1.2 g of stearic acid was dissolved in ethanol with agitation at 220 rpm for 15 min to make a 40% solution. The solution was diluted with distilled water following homogenization. Then, the solution was brought into contact with 50 g of pre-dried sand at a ratio of 22.5 g of water to 3 mL of ethanol, and was stirred for 5 min for a uniform coat. Finally, the coated sand was oven-dried at 80 °C for 3 days to enhance adhesion and to stabilize the hydrophobic film.

### 2.3. Reaction Mechanisms

1. Dissolution: C_17_H_35_COOH dissolves in C_2_H_6_O through hydrogen bonding between ethanol’s hydroxyl group and the carboxylic acid group of stearic acid.

$$C_{17}H_{35}COOH\overset{Ethanol}{\to }Homogeneous\;Solution$$

2. Micelle Formation: Upon the addition of water, stearic acid becomes amphiphilic and forms micelle-like structures with hydrophilic carboxylic groups associated with water, while the hydrophobic alkyl chains (C_17_) orient away.

$${SiO}_{2}-OH+C_{17}H_{35}COOH\overset{Adsorption}{\to }{SiO}_{2}-OOC-C_{17}H_{35}$$

3. Hydrogen Bonding to Sand: The low-energy solution is poured onto the silica sand surface, where hydrogen bonds form between the -OH groups on the sand and the carboxylic groups of stearic acid.

$${SiO}_{2}-OH+C_{17}H_{35}COOH\overset{Drying}{\to }{SiO}_{2}-OOC-C_{17}H_{35}+H_{2}O$$

**Figure 3:**
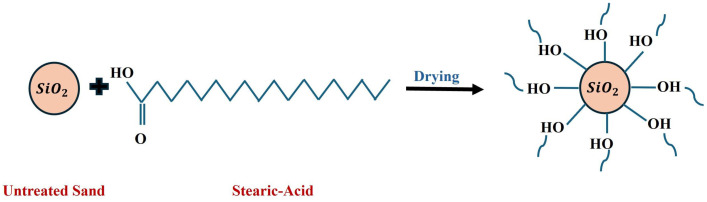
Schematic representation of the formation of SACS.

Figure 3 illustrates the interaction between untreated silica sand and stearic acid molecules during the coating process.

### 2.4. Experimental Setup

The following materials were used: a carbonate–silicate sand mixture from the northern region of Kazakhstan with a particle size of 0.1–800 µm was used; 95% purity C_17_H_35_COOH purchased from Sigma Aldrich (St. Louis, MO, USA) was used as the coating material; C_2_H_6_O and distilled water were used during coating. Additionally, “Terra Vita, Zhivay Zemlya” soil was employed, consisting of 77% high-quality peat with different decomposition degrees, 10% biohumus, 5% washed sand, and 8% perlite. Limestone or dolomite flour was applied to neutralize the acidity of the peat to achieve a pH value of 6.0–6.5. Water penetration into the stearic acid (STA) base sample was restricted owing to the hydrophobicity of SACS. Further, the base sample of STA demonstrated better water-saving ability. Though water in the “Soil” sample was fully evaporated within six days, the base sample of STA held moisture for up to 10 days. This discovery highlights the capacity of SACS in reducing water loss for agricultural purposes.

Even though the results seemed promising, some possible drawbacks were observed. Hydrophobic coatings, especially ones with stearic acid, can degrade in organic solvents with the capability to dissolve stearic acid, which will render them useless [36]. Once the hydrophobic layer is lost, the surface energy will be greater, and the high adhesion of impurities will affect the coating’s performance in corrosion protection [37]. Acid rain reduces the long-term effect of hydrophobic coatings because acidity drives some degradation processes in materials [38]. Earlier studies have documented that the effects of environmental degradation by contact with chemicals reduce performance and raise maintenance expenses for the surfaces under coatings [39]. Three samples were prepared:

1. **Soil (Sample A):** Universal soil only.
2. **Simple Sand Soil:** Untreated sand mixed with universal soil.
3. **STA Base Sample:** Stearic-acid-coated sand mixed with universal soil.

**Figure 4:**
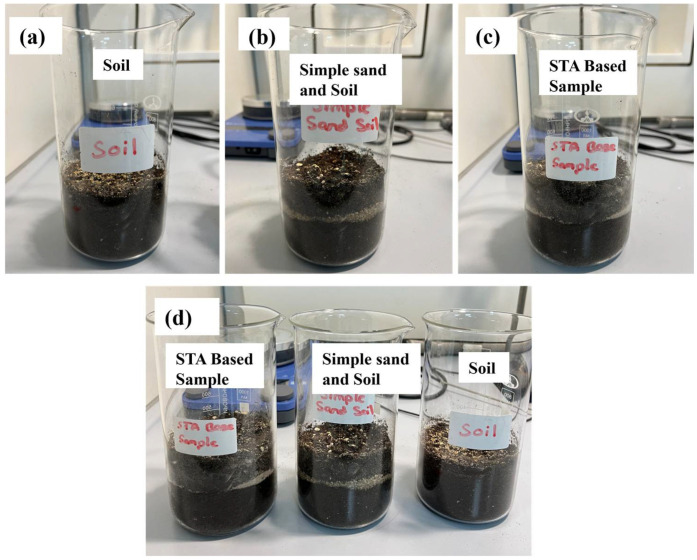
Experimental samples: (**a**) Soil (universal soil only), (**b**) Simple Sand Soil (untreated sand + universal soil), (**c**) STA Base Sample (stearic-acid-coated sand + universal soil), and (**d**) all samples side by side.

**Figure 5:**
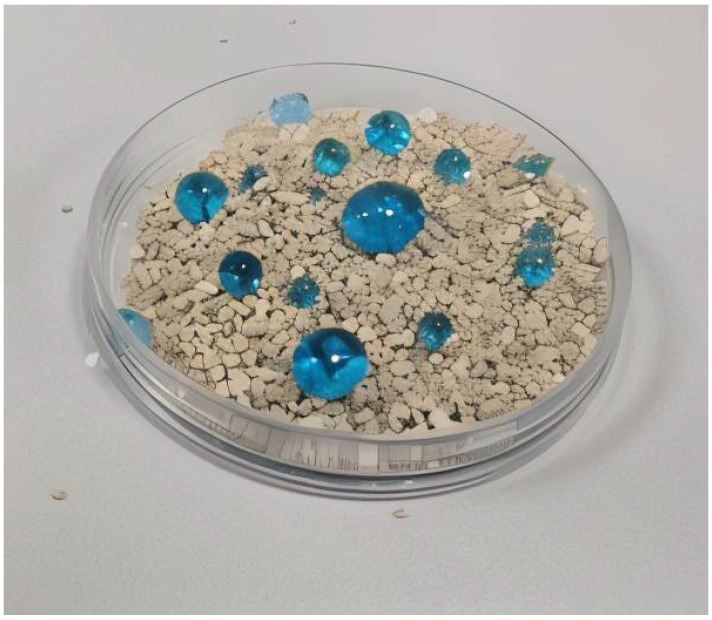
Demonstration of the superhydrophobic properties of SACS.

Samples were placed in separate, labeled glass jars. Hydrophobicity, water retention, and water management characteristics were evaluated. It was observed that water penetration in the STA Base Sample was deterred by the hydrophobic SACS, and water retention lasted up to 10 days, compared with complete evaporation in the Soil sample within 6 days. Potential drawbacks include degradation of the hydrophobic coating in organic solvents or when exposed to acidic conditions. Figure 4 shows the experimental samples and Figure 5 displays the superhydrophobic characteristic of SACS with water droplets spherical in shape.

## 3. Results

### 3.1. Characterization

In order to demonstrate the superhydrophobic properties of SACS, structural, physical, and chemical characterizations were conducted. The XRD technique was employed to investigate the crystal structure and phase composition of the coating, which provides particularly useful information on the crystallinity and integrity of the structure. SEM was employed to observe the surface morphology and microstructure of the coated sand, depicting the homogeneity and effectiveness of the stearic acid layer. Hydrophobicity was quantitatively assessed through contact-angle measurement with a goniometer (OSA-15EC, DataPhysics Instruments GmbH, Filderstadt, Germany) to prove the effectiveness of this coating in water repellency.

**Figure 6:**
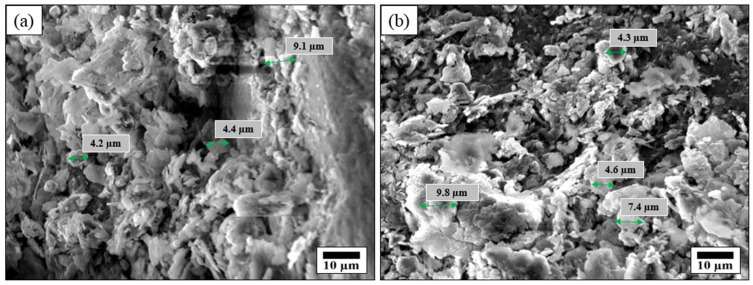
SEM images of SACS display homogeneous stearic acid coating with micro-scale aggregates (4–10 μm) leading to hierarchical roughness, a prerequisite for inducing superhydrophobicity (WCA > 150°). (**a**) SEM image showing uniformly distributed micro-aggregates with no visible cracks or defects, suggesting strong adhesion. (**b**) SEM image from a different area of the same sample, further confirming the consistent morphology and aggregate size distribution. Uncoated regions or cracks are not observable, indicating good adhesion.

Figure 6a,b present SEM images of SACS at different magnifications, showing that the surface of the sand particles is uniformly coated with stearic acid deposits in the form of clusters and aggregates with diameters ranging from approximately 4 to 10 μm. These agglomerates, which encompass probable portions of embedded silica grains, develop very irregular and textured morphology, with protrusions or “islands” that induce a hierarchical micro-scale roughness. Such morphological complexity is crucial for superhydrophobicity, as the textured surface topography creates additional interfaces where water droplets can dwell and thereby avoid direct wetting of the substrate. While the micro-scale features are heterogeneous in nature, the coating is even with minor patches of non-coated material, suggesting that the fabrication procedure effectively resulted in an even layer of stearic acid. The various size measurements (e.g., 4.2 μm, 4.4 μm, 7.4 μm, 9.1 μm, and 9.8 μm) indicate that these clusters overlap or coalesce to form a coating several micrometers thick in certain regions, a typical characteristic of granular material coatings. The hierarchical micro-roughness induced by these overlapping clusters is also the cause of the high water repellency due to the fact that water droplets are forced to rest on the elevated topography rather than spreading within the valleys, thus creating high contact angles (WCA > 150°). In addition, the presence of no discernible cracks or delaminated areas in the photographs contradicts the expectation of there being no stearic acid on the silica surface. This is due to their strong adhesion, and suggests that the material has been stabilized, likely by hydrogen bonding or similar interfacial interaction with the acid carboxylic groups and hydroxyl sites in the sand, as suggested by complementary FTIR and Raman investigations. The SEM analysis presents definite morphological proof of the homogeneous and effective deposition of the stearic acid on the sand, forming a water-repellent, long-lasting layer crucial for improving water management in agricultural conditions.

**Figure 7:**
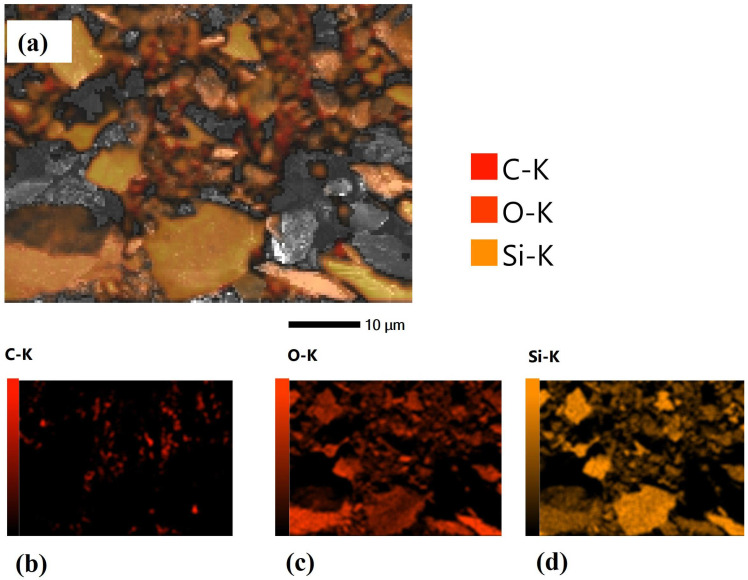
EDS mapping of SACS showing elemental distribution: (**a**) Composite elemental map displaying overlapping distributions of carbon (C–K), oxygen (O–K), and silicon (Si–K); (**b**) Elemental map showing the distribution of carbon (C–K), indicating the presence of organic stearic acid coating; (**c**) Elemental map showing the distribution of oxygen (O–K), primarily from both the sand substrate and organic layer; (**d**) Elemental map showing the dis-tribution of silicon (Si–K), confirming the presence of the silica substrate.

Figure 7 presents the EDS mapping of SACS and gives an overall description of the elemental distribution on the sample surface. The mapping clearly shows the presence of carbon (C-K), oxygen (O-K), and silicon (Si-K), each with a specific color representation. The carbon mapping shown in Figure 7b shows a uniform layer of carbon, thus confirming the successful application of stearic acid on the silica sand. The oxygen map (Figure 7c) reveals a homogeneous distribution of oxygen, thereby corroborating the homogeneous deposition of the stearic acid layer. Meanwhile, the silicon map (Figure 7d) brings out the fact that there is an underlying silica sand substrate, a sign that the coating process does not undermine the structural integrity of the silica particles. These maps collectively provide us with strong evidence of the successful functionalization of the sand surface. The uniform distribution of these components proves that stearic acid has been evenly coated onto the silica sand, which leads to the formation of a stable hydrophobic barrier. Such a property is fundamental for enhancing water retention and reducing evaporation in soils used for agriculture, therefore highlighting the potential of SACS as an environmentally friendly method for water management in dry regions.

**Figure 8:**
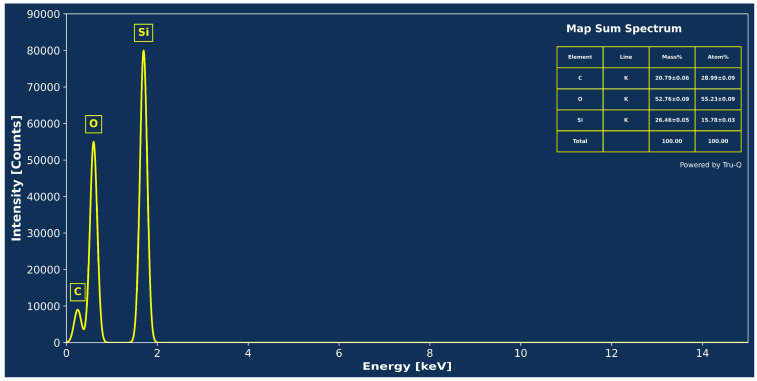
EDS spectrum and quantitative elemental analysis of SACS.

Figure 8 shows the EDS spectrum of the SACS, showing characteristic Kα emission peaks for carbon (C–K, ~0.3 keV), oxygen (O–K, ~0.5 keV), and silicon (Si–K, ~1.7 keV), and the quantitative elemental analysis. The prominent O–K and Si–K peaks confirm the important contribution of the silica substrate, while the prominent C–K signal provides conclusive evidence for the organic layer made up of stearic acid. The mass and atomic percentage measurements indicate that almost one-fifth of the sample’s total mass is from carbon-based coating materials, which is consistent with the existence of a conformal film of stearic acid coating the sand particles. The close concordance achieved between the predicted and measured elemental ratios, with a compatible ratio of 0.1409, confirms the validity of the measurement process.

**Figure 9:**
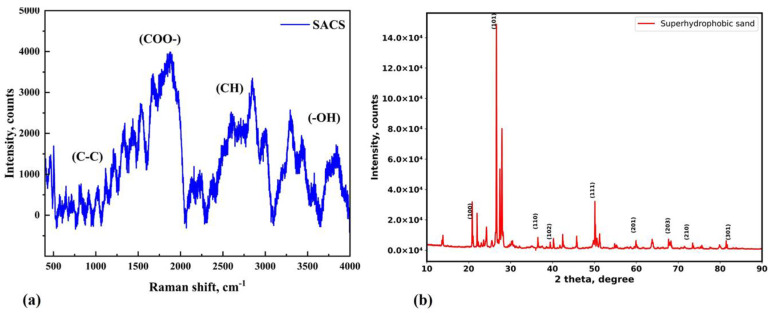
(**a**) Raman spectroscopy confirming the presence of functional groups from stearic acid; (**b**) XRD pattern indicating the crystalline integrity of the sand substrate after coating.

Figure 9a shows the Raman spectrum of the SACS and provides robust vibrational characteristics that indicate successful surface modification. The spectrum presents a broad asymmetric peak in the 1600 to 1700 cm^−1^ range, which is due to the asymmetric stretching vibrations of stearic acid’s carboxylate groups (COO^−^). The asymmetry observed in this region of the spectrum may be due to the superimposition of the effects of vibrations in the case of surface hydroxyls or adsorbed water, along with hydrogen bonding effects that prevail between the silica surface and stearic acid, as reported in earlier works [40]. Furthermore, characteristic hydrocarbon chain bands are seen between 2800 and 3000 cm^−1^ due to C–H stretching vibrations. These bands confirm the presence of long aliphatic chains from stearic acid, which are accountable for the material’s hydrophobicity.

The broad band in the 3000–3700 cm^−1^ range is due to O–H stretching vibrations. The band is a function of the surface hydroxyl groups (silanol, Si–OH) of the silica substrate and perhaps of residual moisture. In agreement with Bansal and Doremus (1986), silanol groups are typically characterized by stretching bands in this range, with specific positions depending on hydrogen bonding environments [41]. These spectral features collectively confirm the effective coating of silica sand with stearic acid, making it superhydrophobic for immediate application in water-retentive agricultural technology.

Figure 9b shows the XRD pattern of the SACS sample, which is very important in determining the structural integrity of the sand substrate after coating. The XRD pattern exhibits sharp and well-defined peaks at 2θ values for crystallographic planes such as (101), (110), (102), (111), (201), (203), (210), and (301). These peaks are characteristic of the crystalline nature of silica sand. That these peaks are maintained, with no measurable broadening or shifting, demonstrates that the inherent crystallinity of the sand remains intact after the stearic acid coating has been applied. This preservation is crucial since it means that the coating process does not result in any amorphization or degradation of the sand particles, thereby maintaining the mechanical and structural integrity of the material for practical applications.

The combined Raman and XRD analyses provide conclusive evidence that the stearic acid is successfully deposited onto the sand particles. The Raman spectrum confirms the chemical character of the coating, with the presence of both the aliphatic chains and the carboxylate functions of the stearic acid, and the XRD pattern confirms that the coating process preserves the crystalline structure of the silica substrate. Cumulatively, these observations facilitate the formulation of a stable hydrophobic coating that is key to reducing water loss and enhancing water retention within agricultural systems, thereby highlighting the potential of SACS as an efficient material for improved agricultural water management.

**Figure 10:**
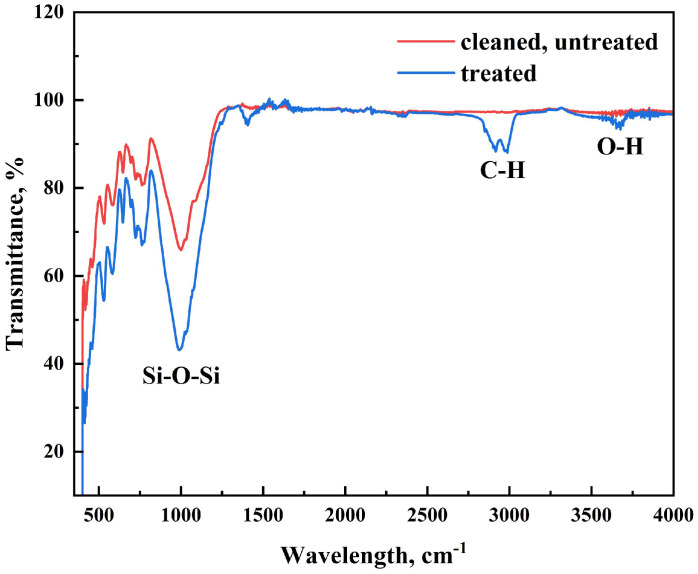
FTIR spectra of uncoated and stearic-acid-coated sand, with focus on appearance of C–H, C=O, and O–H bands confirming successful coating.

The FTIR spectrum (Figure 10) clearly indicates differences between the untreated sand and the coated sand with stearic acid, verifying the successful surface modification. The untreated sand spectrum is characterized by a broad absorption band between 500 and 1200 cm^−1^, typical of the Si–O–Si stretching vibrations of silica. In the coated sand spectrum, there are additional peaks that are not present in the untreated sample. Most importantly, the appearance of the aliphatic C–H stretch near 2850 cm^−1^ and 2920 cm^−1^ confirms the occurrence of stearic acid’s long hydrocarbon chains. There is also a prominent carbonyl (C=O) stretch around 1700 cm^−1^, which is representative of the carboxylic acid functional group contained in stearic acid. A broad band at about 3400 cm^−1^ also signals the presence of O–H groups, potentially due to surface-adsorbed water or hydrogen bonding. Generally, the appearance of these additional peaks in the FTIR spectrum of the coated sand signifies the successful adsorption of stearic acid, hence altering the surface chemistry of the sand towards potential agricultural applications due to its enhanced hydrophobicity and changed adsorption properties.

### 3.2. WCA Measurement

**Figure 11:**
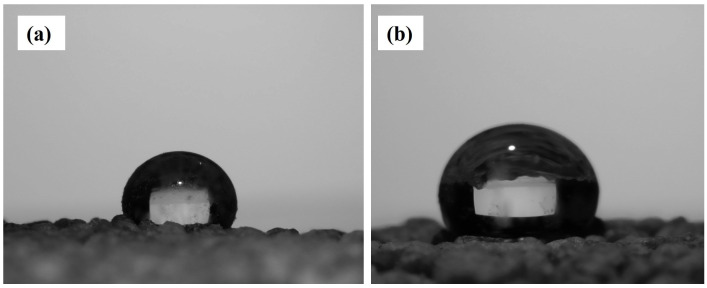
Images of water droplets on SACS surfaces showing WCAs of (**a**) 152.7° and (**b**) 151.2°.

WCA measurements were performed using a goniometer (OSA-15EC) and analyzed with ImageJ (version 1.53e). Figure 11 shows water droplets on the SACS surface with measured WCAs of 152.7° and 151.2°, confirming the superhydrophobicity of the coating.

**Figure 12:**
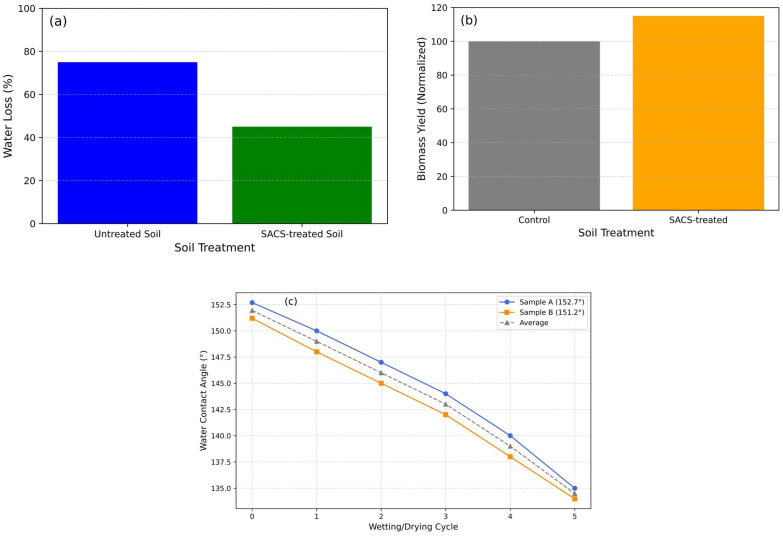
Performance evaluation of SACS in terms of soil moisture retention and plant biomass yield. (**a**) Water loss from untreated vs. SACS-treated soil after 10 days under controlled laboratory conditions. (**b**) Biomass yield comparison between control and SACS-treated soil in initial pot trials. (**c**) Water contact angle (WCA) reduction of stearic-acid-coated sand over five wetting/drying cycles, showing progressive degradation of surface hydrophobicity.

We approximated, in our tests, the stearic acid coating on 50 g of sand to be approximately 6 μm thick—calculated from the consumption of 1.2 g of stearic acid and an estimation of the total surface area of the sand particles with qualitative verification through SEM—and utilized the coated sand as a surface layer on the soil surface by evenly distributing 50 g across 50 cm^2^, which created a layer thickness of approximately 6–7 mm based on volume calculations assuming a packing density of ~1.5 g/cm^3^. Water evaporation experiments conducted under controlled laboratory conditions (≈25 °C, 40–50% relative humidity, negligible air movement) using a precision balance (±0.01 g accuracy) showed that water loss from untreated soil was approximately 75% after 10 days, compared with approximately 45% for SACS-treated soil (Figure 12a). Moreover, initial pot trials showed an approximate 10–20% greater biomass yield in the SACS-treated soil compared to the control (Figure 12b). The long-term stability of the stearic acid coating was examined by exposing it to five successive cycles of wetting/drying, and WCAs were measured after each cycle by a goniometer (OSA-15EC). As shown in Figure 12c, Sample A and Sample B both exhibited a systematic and consistent reduction in WCA with each cycle. The starting WCAs were 152.7° and 151.2°, respectively—both indicative of superhydrophobicity. Each WCA value reported is the average of five separate measurements from various positions on the sample surface, in an effort to average out potential surface heterogeneity. The standard deviation between the measurements was ±1.5°, a figure within that anticipated for manual droplet analysis in controlled laboratory conditions.

The linearity in the WCA reduction for both samples is indicative of homogeneous patterns of degradation across the tested regions, suggesting homogeneous wear or partial restructuring of the stearic acid film under exposure to moisture. This progressive yet modest loss of hydrophobicity—averaging a ~10–15% loss per five cycles—demonstrates the durability of the coating while highlighting the need for further research into long-term environmental durability.

### 3.3. Validation of Stearic Acid Coating

The successful coating of stearic acid on the sand surface was validated using a combination of structural, chemical, and physical characterization techniques, including SEM, Raman spectroscopy, XRD, and FTIR spectroscopy. The SEM images revealed a smooth and uniformly coated sand surface at low magnification, confirming the effective application of the stearic acid layer. Higher magnification images highlighted the presence of unevenly distributed clusters, enhancing surface roughness and contributing to improved hydrophobicity.

Raman spectroscopy further verified the chemical composition of the coating, with characteristic peaks indicating the presence of long hydrocarbon chains (C-C), carboxylate groups (COO^−^), and alkane stretching vibrations (C-H). A reduction in the -OH peak intensity suggested successful functionalization of the sand surface. XRD analysis confirmed that the crystalline structure of the sand remained unaffected by the coating process, ensuring mechanical and structural integrity.

Finally, FTIR spectra demonstrated characteristic peaks of stearic acid functional groups, including methylene (-CH_2_) stretching, carbonyl (C=O) vibrations, and broad O-H stretching. These results collectively confirm the presence of a uniform and stable stearic acid layer, effectively rendering the sand surface hydrophobic and suitable for water-repellent applications in agriculture.

## 4. Conclusions

This study validated that SACS synthesis and characterization is an intriguing, inexpensive, scalable method for enhancing water management in sandy soils, which is a critical need in arid agricultural regions. Utilizing 1.2 g of stearic acid in an ethanol–water solution to treat 50 g of sand, we achieved a uniform hydrophobic layer with an estimated thickness of 6 μm, as confirmed by SEM showing a homogeneous deposition with micro-scale aggregates of 4 to 10 μm in diameter, which generated the hierarchical roughness essential for superhydrophobicity. WCA analyses also demonstrated the effectiveness of the coating, with average angles of 152.7° and 151.2°, confirming that the material repels water significantly. Under controlled lab conditions (≈room temperature, 40–50% relative humidity), SACS-treated soil experienced significant reductions in the evaporation of water—only an approximate 45% loss compared to approximately 75% in untreated soil over a period of 10 days—thereby extending moisture duration and showing important agronomic benefits. Initial pot trials supported these findings, with 10–20% greater biomass production in plants grown in SACS-treated soil than in control samples. Five repeated cycles of wet–dry durability tests indicated a moderate loss of 10–15% in hydrophobicity, indicating the need for further research into long-term durability under different environmental conditions and potential ecotoxicity. In general, the quantitative results of this study verify that SACS not only effectively decreases water evaporation and increases soil moisture storage, but also possesses promise as a sustainable solution for addressing water scarcity issues in arid agricultural environments.

## Data Availability

The data supporting the conclusions of this article will be made available by the corresponding author upon request.
